# Improving the NO_2_ Gas Sensing Performances at Room Temperature Based on TiO_2_ NTs/rGO Heterojunction Nanocomposites

**DOI:** 10.3390/nano14221844

**Published:** 2024-11-18

**Authors:** Yan Ling, Yunjiang Yu, Canxin Tian, Changwei Zou

**Affiliations:** 1 Key Laboratory of Advanced Coating and Surface Engineering, Lingnan Normal University, Zhanjiang 524048, China; lingyan@lingnan.edu.cn (Y.L.); sidui@lingnan.edu.cn (Y.Y.); cxtian@lingnan.edu.cn (C.T.); 2 Research Center for Engineering Technology in Surface Strengthening of Guangdong Province, Lingnan Normal University, Zhanjiang 524048, China

**Keywords:** TiO_2_ nanotubes, reduced graphene oxide, NO_2_ gas sensing, nanocomposites, room temperature sensors

## Abstract

The development of energy-efficient, sensitive, and reliable gas sensors for monitoring NO_2_ concentrations has garnered considerable attention in recent years. In this manuscript, TiO_2_ nanotube arrays/reduced graphene oxide nanocomposites with varying rGO contents (TiO_2_ NTs/rGO) were synthesized via a two-step method for room temperature NO_2_ gas detection. From SEM and TEM images, it is evident that the rGO sheets not only partially surround the TiO_2_ nanotubes but also establish interconnection bridges between adjacent nanotubes, which is anticipated to enhance electron–hole separation by facilitating electron transfer. The optimized TiO_2_ NTs/rGO sensor demonstrated a sensitive response of 19.1 to 1 ppm of NO_2_, a 5.26-fold improvement over the undoped TiO_2_ sensor. Additionally, rGO doping significantly enhanced the sensor’s response/recovery times, reducing them from 24 s/42 s to 18 s/33 s with just 1 wt.% rGO. These enhancements are attributed to the increased specific surface area, higher concentration of chemisorbed oxygen species, and the formation of p-n heterojunctions between TiO_2_ and rGO within the nanocomposites. This study provides valuable insights for the development of TiO_2_/graphene-based gas sensors for detecting oxidizing gases at room temperature.

## 1. Introduction

As a harmful air pollutant, nitrogen dioxide (NO_2_) has been responsible for significant environmental issues, including acid rain and photochemical smog, which pose serious risks to human health [1,2]. Therefore, the on-line and real-time monitoring of NO_2_ leakage is crucial for protecting both public health and environmental safety. The development of energy-efficient, sensitive, and reliable gas sensors for monitoring NO_2_ concentrations has obtained considerable attention in recent years [3,4,5]. Metal oxides are well known for their excellent adsorption capacity, catalytic activity, and thermodynamic stability and are widely utilized in gas sensor applications [6]. Numerous metal oxides have been investigated as potential gas sensors, including ZnO, SnO_2_, In_2_O_3_, WO_3_, Fe_2_O_3_, and TiO_2_ [6,7,8,9,10,11,12]. However, the performance of NO_2_ gas sensors based on metal oxides remains unsatisfactory due to several limitations, such as high operating temperatures, elevated energy consumption, and poor reproducibility [13,14]. Notably, most metal oxide gas sensors require operation at high temperatures ranging from 200 to 400 °C. This not only makes them unsuitable for detecting gases that may contain explosive substances but also poses cost challenges for commercial applications. The prolonged response and recovery times, along with the low sensitivity at room temperature, represent significant bottlenecks for the practical applicability of TiO_2_-based gas sensors. To address these issues, it is essential to incorporate or integrate TiO_2_ with hybrid nanocomposite materials such as graphene compounds and metal nanoparticles. This approach has been widely regarded as an effective strategy to enhance gas sensing performance.

Due to the introduction of oxygen functional groups and surface defects that serve as active sites for gas adsorption, graphene oxide (GO) and reduced graphene oxide (rGO) are increasingly recognized as suitable materials for room temperature gas sensing [15]. However, due to the limitations in gas sensor fabrication associated with the low defect density of graphene, numerous researchers have sought to develop gas sensors utilizing reduced graphene oxide (rGO), which possesses a higher defect density [16,17]. The development of low cost, transparent, and flexible rGO-based sensors for detecting harmful gases at very low concentrations is significantly important yet still challenging [18]. The TiO_2_ nanoparticles/reduced graphene oxide (TiO_2_ NPs/rGO) composite demonstrated a significant gas response (~14.9%), which is 4.57 times higher than that of pristine counterparts, along with excellent selectivity, high sensitivity, rapid response and recovery times, as well as remarkable repeatability towards nitrogen dioxide (NO_2_) at a concentration of 100 ppm at room temperature [19]. The anti-humidity sensing performance of Pt/GO/TiO_2_ is improved by increasing the thickness of the GO interlayer. Remarkably, the diode with a GO areal loading of 0.969 mg cm^−2^ exhibits a response retention rate (R_RH95%_/R_dry_) of nearly 100% at 298 K [20]. A variety of high-performance and low-temperature gas sensors based on rGO hybrids have been reported. A straightforward one-pot microwave-assisted hydrothermal method has been employed to synthesize SnO_2_/rGO composites, resulting in a significant reduction in the response and recovery times from 39.2/54.7 min to just 6.5/1 min, with an impressive detection limit as low as 50 ppb [21]. Under UV irradiation from an LED, a sensor utilizing graphene/TiO_2_ nanoparticles demonstrated a detection limit of approximately 50 ppb for NO_2_ at room temperature [22]. NO_2_ gas sensors based on a ZnO-rGO hybrid showed improved sensitivity and faster response and recovery times [23]. Furthermore, a low-operating-temperature NO_2_ gas sensor based on rGO/SnS_2_ has shown remarkable selectivity and reversibility towards NO_2_, achieving a low detection limit of 0.6 ppm with a response rate of 9.8% at 80 °C [24]. Moreover, when exposed to 1 ppm NO_2_ at room temperature, CuO/rGO hybrids displayed a sensitive response quantified at around 14 [25].

In this manuscript, we fabricated a highly efficient room temperature NO_2_ sensor utilizing TiO_2_ nanotubes/reduced graphene oxide (TiO_2_ NTs/rGO) nanocomposites. In addition, the fabricated gas sensor exhibits a highly sensitive response and excellent selectivity towards NO_2_ gas, and the mechanism underlying the gas sensing performance was also investigated.

## 2. Experimental Details

### 2.1. Synthesis of Materials

**Figure 1:**
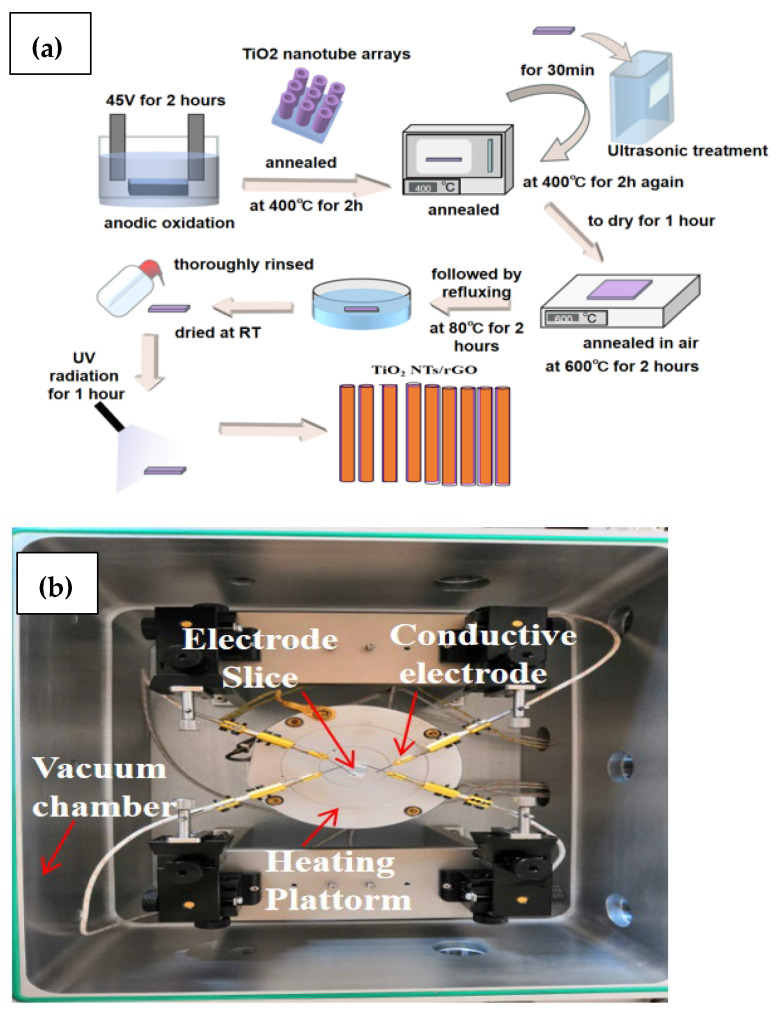
Schematic of synthesis process (**a**) and photograph of NO_2_ detection testing system (**b**).

All chemical reagents were of analytical grade (Beijing Chemical Co., Ltd., Beijing, China) and utilized without further purification. TiO_2_ nanotube arrays were synthesized through the anodization of titanium foil (99.99%) at a voltage of 45 V for a duration of 2 h. The electrolyte was composed of ethylene glycol (99.99%), 0.3 M ammonium fluoride (NH_4_F, 99%), and 2 vol% water (H_2_O). Following anodization, the samples were annealed at 400 °C for 2 h, after which they were sonicated for 30 min and subjected to another round of annealing for 2 h. Then, the samples were removed from the bath and allowed to dry at room temperature for 1 h before being further annealed at 600 °C for 2 h in a furnace under air atmosphere. Subsequently, the samples were immersed in a solution containing 3-aminopropyl triethoxysilane and ethanol, followed by refluxing at 80 °C for 2 h. The TiO_2_ nanotubes were then thoroughly rinsed with ethanol and deionized water before being dried at room temperature. The reduction of graphene oxide (GO) into reduced graphene oxide (rGO) was accomplished by exposing GO suspension to UV radiation for 1 h. The electrostatic interaction between positively charged nanotubes and negatively charged rGO facilitates the adhesion of graphene derivatives onto the surface of the nanotubes [26]. The theoretical weight percentages of rGO within the TiO_2_ NTs/rGO nanocomposites were calculated to be approximately 0.5 wt%, 1 wt%, and 3 wt%. For clarity, these TiO_2_ NTs/rGO nanocomposites will henceforth be referred to as containing either 0.5 wt%, 1 wt%, or 3 wt% rGO in subsequent figures. Figure 1a displays a schematic of the synthesis process.

### 2.2. Characterization

The energy dispersive spectrometry (EDS) and field emission scanning electron microscopy (FESEM) images were acquired using a JEOL JSM-7500F microscope operating at 15 kV (JEOL, Japan). For the X-ray powder diffraction (XRD) analysis, we utilized X-ray diffractometer (XRD; D8, Karlsruhe, Germany) at a scanning rate of 0.02 s^−1^. The Raman spectroscopy analysis was performed using the RENISHAW INVIA Micro-Raman spectrometer (Renishaw, UK). The X-ray photoelectron spectroscopy (XPS, Kratos XSAM800, Kratos Ltd., Manchester, Britain) was used to examine the chemical bonding states with Mg K_a_ excitation. Transmission electron microscopy (TEM) and high-resolution transmission electron microscopy (HR-TEM) were conducted on a JEOL JEM-2100F microscope operating at an accelerating voltage of 200 kV. The specific surface area was estimated using the Brunauer–Emmett–Teller (BET) (BET, 3H-2000ps4, China) equation based on nitrogen adsorption isotherms, following prior degassing of the sample under vacuum at 120 °C.

### 2.3. Fabrication and Measurement of the Gas Sensor

The photograph and structure of the NO_2_ detection equipment are presented in Figure 1b [27,28]. The fabrication process can be described as follows: First, a suitable amount of the as-grown TiO_2_ NTs/rGO powder was thoroughly mixed with deionized water to create homogeneous slurry. This slurry was then carefully coated onto an alumina tube using a small brush to form a sensing film. A pair of Au electrodes was installed at each end of the tube, with each electrode connected to a pair of Pt wires. After allowing it to dry in air at room temperature, the device underwent annealing at 200 °C for 2 h to eliminate any residual water. Finally, a Ni-Cr alloy coil was inserted into the alumina ceramic tube to serve as a heater. The operating temperature is controlled by adjusting the heating current supplied to the ceramic heater. The response of the sensor is defined as S = R_g_/R_a_, where R_a_ and R_g_ represent the electrical resistance of the gas sensor in air and in NO_2_, respectively. The response time or recovery time was defined as the time taken for 90% resistance variation.

## 3. Results and Discussion

### 3.1. Structural and Morphological Characteristics

**Figure 2:**
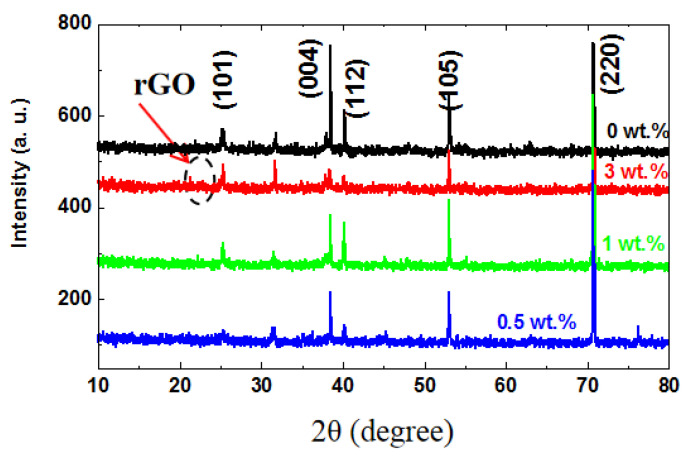
XRD patterns of TiO_2_ NTs/rGO nanocomposites grown with different rGO contents.

Figure 2 presents the XRD patterns of the TiO_2_ NTs/rGO nanocomposites synthesized with varying rGO contents. The XRD pattern for undoped TiO_2_ nanotubes displays very sharp diffraction peaks, all of which can be confidently assigned to the anatase TiO_2_ phase (JCPDS Card No. 21-1272). It is important to note that the incorporation of reduced graphene oxide does not alter the original crystal structure of TiO_2_. All diffraction peaks in the nanocomposites are observed at nearly identical 2θ positions when compared to those of undoped TiO_2_. However, with an increase in rGO doping, there is a noticeable decrease in the intensity of the diffraction peaks for TiO_2_ NTs/rGO nanocomposites, which can be attributed to an excess formation of nucleation centers [29]. Additionally, it is interesting to observe from the XRD pattern of TiO_2_ NTs/rGO with an rGO content of 3 wt.% that a weak peak appears between 23° and 26°, which corresponds to the (002) plane of rGO [30,31].

**Figure 3:**
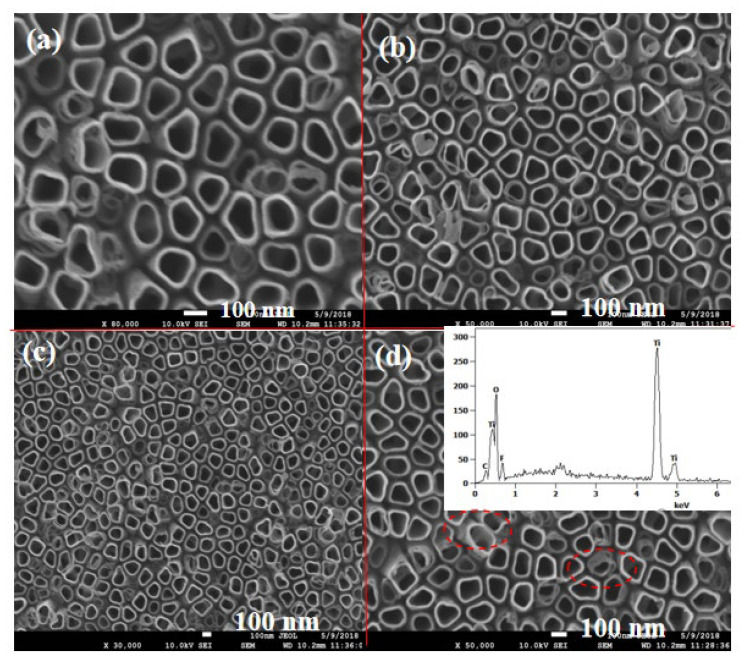
SEM images of TiO_2_ NTs/rGO nanocomposites with rGO contents of 0 wt.% (**a**), 0.5 wt.% (**b**), 1 wt.% (**c**), and 3 wt.% (**d**), respectively. Inset of Figure 3d shows the EDX spectrum of TiO_2_ NTs/rGO nanocomposites with rGO contents of 3 wt.%. The red circles in Figure 3d illustrate the structure of rGO surrounding the TiO_2_ nanotubes.

As illustrated in Figure 3a, the TiO_2_ nanotubes are characterized by vertically arranged structures with an average diameter ranging from approximately 80 to 120 nm. For the TiO_2_ NTs/rGO nanocomposites with rGO concentrations of 0.5 and 1 wt.%, graphene sheets are not distinctly visible in Fig. 3b and 3c. However, for those with rGO contents of 3 wt.%, it is evident that the rGO sheets not only partially surround the TiO_2_ nanotubes but also establish interconnection bridges between adjacent nanotubes (Figure 3d). This connectivity through the rGO layer is anticipated to enhance electron–hole separation by facilitating electron transfer from TiO_2_. In addition, electrons traveling along the graphene layer may interact with adsorbed NO_2_ molecules as well. To confirm the presence of graphene within these composites, we conducted an EDS analysis on samples with a rGO content of 3 wt.%. The EDX spectrum presented in the inset of Figure 3d reveals peaks corresponding to titanium (Ti), oxygen (O), and carbon (C) elements.

**Figure 4:**
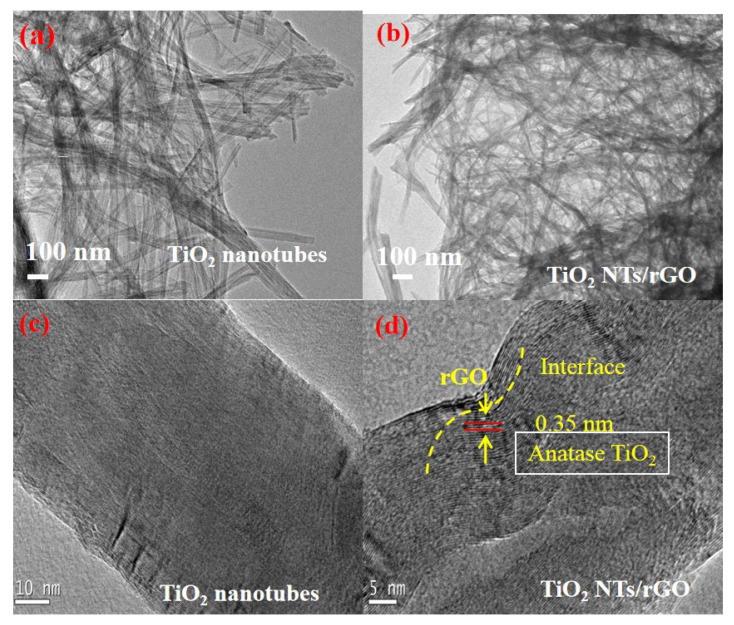
TEM (**a**,**b**) and high-resolution TEM (**c**,**d**) images of TiO_2_ nanotubes (**a**,**c**) and TiO_2_ NTs/rGO nanocomposites (**b**,**d**) with rGO contents of 1 wt.%.

As can be seen in the TEM images shown in Figure 4a,b, there is no significant difference in diameter and length between TiO_2_ nanotubes and TiO_2_ NTS/rGO nanocomposites. To gain deeper insights into the nature of the interface, we conducted HR-TEM analysis, with the corresponding image presented in Figure 4d. When compared to the undoped TiO_2_ nanotubes shown in Figure 4c, it becomes evident that thin layers are wrapped around the outer surface of the TiO_2_ nanotubes, indicating successful modification by rGO. The observed lattice spacing of 0.35 nm for the TiO_2_ nanotubes corresponds to the (110) orientation of anatase phase TiO_2_. The layer material in contact with TiO_2_ is rGO, which forms a close-contact interface with the surface of these nanotubes, which is believed to facilitate electron transmission. However, it is important to note that any observed inhomogeneity within the nanocomposites may arise from limitations associated with chemical processes or reaction times, or the sample preparation method for TEM.

### 3.2. Raman and XPS Characteristics

**Figure 5:**
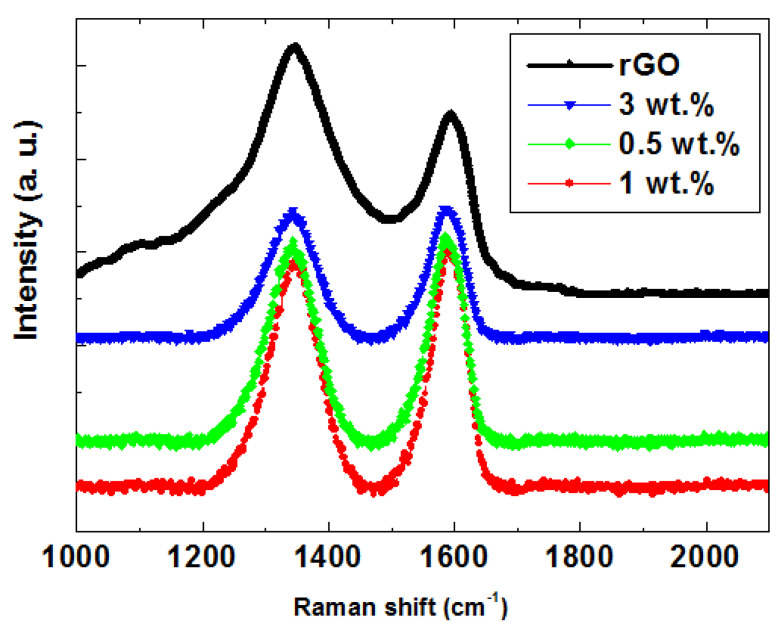
Raman spectra of TiO2 NTs/rGO nanocomposites with different rGO contents.

The Raman spectra of TiO_2_ NTs/rGO nanocomposites, as illustrated in Figure 5, reveal two prominent peaks corresponding to the D and G bands of graphene. The G band provides valuable information regarding the in-plane vibrations of sp^2^ bonded carbon atoms [32], while the D band is associated with some sp^3^ defects present in rGO [33]. The characteristics of Raman modes of anatase phase appeared at 146, 400, 518, and 636 cm^−1^ for pristine TiO_2_, and these peaks are well matched with E_g_, B_1g_, A_1g_ + B_1g_, and Eg modes of the anatase TiO_2_ phase [34]. As depicted in Figure 5, the G band for TiO_2_ NTs/rGO composites appears at 1597 cm^−1^, which is quite close to that of pristine graphene (1580 cm^−1^). The slight red shift observed in the G band for TiO_2_ NTs/rGO nanocomposites compared to GO (1591 cm^−1^) suggests a restoration of the π-π systems within graphene during the chemical reaction. Additionally, it is widely recognized that the *I*_D_/*I*_G_ ratio serves as a valuable indicator of crystal quality within graphite structures. Our calculations indicate that the *I*_D_/*I*_G_ ratio for 3 wt.% TiO_2_ NTs/rGO (1.26) exceeds that of 0.5 wt.% TiO_2_ NTs/rGO (1.14), signifying a stronger D band signal and thus confirming successful incorporation of rGO into these nanocomposites. Additionally, spectral characteristics suggest an increase in defect point density on the resulting rGO sheets, which may serve as ideal adsorption–desorption sites for TiO_2_ nanotubes [31].

**Figure 6:**
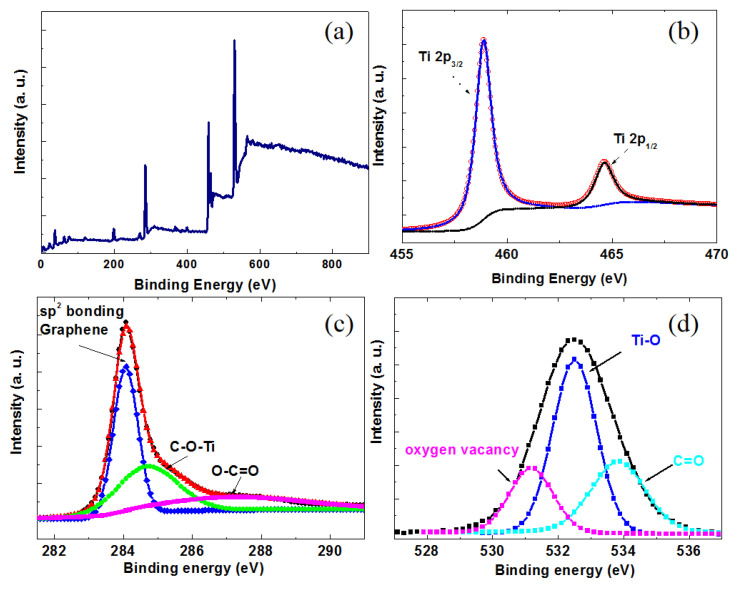
XPS spectra of TiO_2_ NTs/rGO nanocomposite with rGO contents of 1 wt.%. (**a**) Full scan. (**b**) Ti 2p. (**c**) C 1s. (**d**) O 1s.

The chemical states of the elements were carefully analyzed using XPS and are illustrated in Figure 6. The sharp peaks observed in the full scan spectra indicate a clear presence of C, O, and Ti elements for TiO_2_ NTs/rGO nanocomposites. The TiO_2_ NTs/rGO nanocomposite with a rGO content of 1 wt.% exhibits the best gas sensing performance. Therefore, we selected this sample for XPS characterization. In Figure 6b, the Ti 2p_3/2_ and Ti 2p_1/2_ peaks are located at binding energies of 458.9 and 464.8 eV, which correspond to the values for Ti^4+^ in TiO_2_ [35]. As shown in Figure 6c, the high-resolution C 1s peak was accurately fitted with three distinct components. The binding energy at 284.3 eV, 285.1 eV, and 287.5 eV can be attributed to C-C bonds (sp^2^ hybridized carbon) from rGO, C-O-Ti bonds and O-C=O species, respectively [36]. The fitted peak at 285.1 eV corresponding to the C-O-Ti bond further confirms that a chemically bonded heterostructure has indeed formed between TiO_2_ NTs and rGO [37]. This heterostructure facilitates close contact between TiO_2_ nanotubes and rGO, thereby promoting effective electron transfer. In Figure 6d, we present the high-resolution XPS spectrum for O 1s, which can be resolved into three Gaussian peaks representing different types of oxygen species. The three peaks can be respectively attributed to oxygen vacancy in defective TiO_2_, lattice oxygen species (Ti-O bands)_,_ and chemisorbed or dissociated oxygen species (C=O) [38]. The increase in oxygen vacancies within defective TiO_2_ suggests that there are more active sites available in the TiO_2_ NTS/rGO nanocomposites, which is advantageous for gas adsorption and reaction. Additionally, the rise in C=O bonds in these nanocomposites indicates that the oxygen species adsorbed on the surface can participate effectively in surface redox reactions, leading to significant changes in sensor resistance. Therefore, by incorporating rGO, the TiO_2_ NTs/rGO nanocomposite demonstrates a remarkable ability to adsorb ionized oxygen, contributing to its potential for high-performance gas sensing applications [39].

### 3.3. Gas Sensing Properties

**Figure 7:**
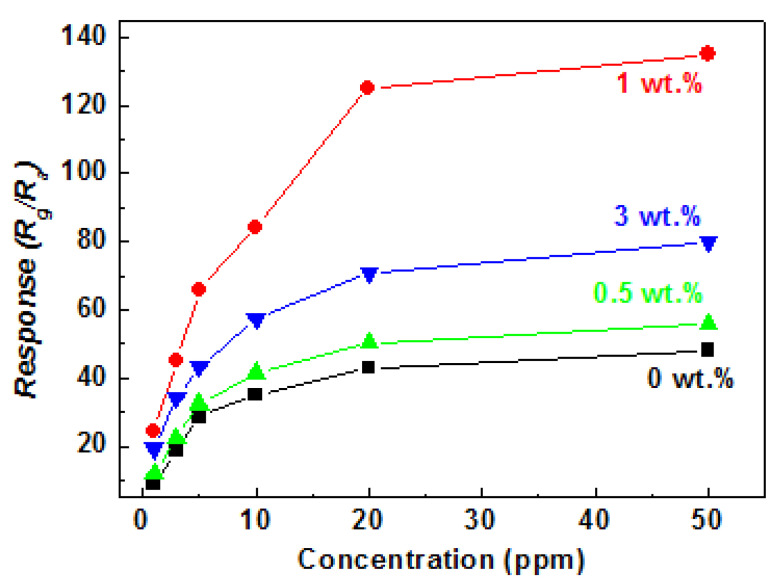
The response value of the sensor based on TiO_2_ NTs/rGO composites vs. NO_2_ concentration at room temperature.

The relationship between the response and NO_2_ concentration for the sensor based on TiO_2_ NTs/rGO nanocomposites at room temperature is illustrated in Figure 7. It can be observed that the response increases with NO_2_ concentration, ranging from 1 ppm to 50 ppm. Notably, the TiO_2_ NTs/rGO nanocomposite with contents of 1.0 wt.% exhibited the highest response value, reaching an impressive 138 at a NO_2_ concentration of 50 ppm. The limit of detection was determined to be 1 ppm, and it is worth mentioning that the sensor demonstrated a remarkable response of 19.1 for just 1 ppm NO_2_ when utilizing the TiO_2_ NTs/rGO composite with rGO contents of 1.0 wt.%.

**Figure 8:**
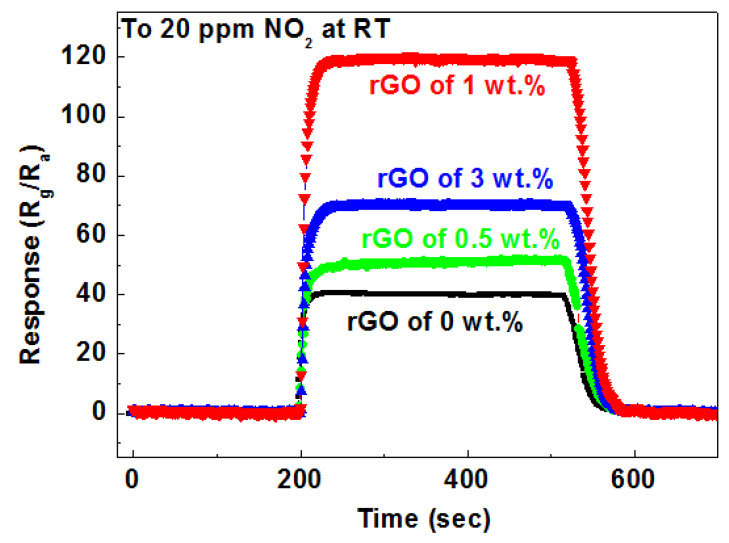
The dynamic response transients of the sensor based on TiO_2_ NTs/rGO nanocomposites to 20 ppm NO_2_ at room temperature.

The response and recovery curves of the sensor to 20 ppm NO_2_ are presented in Figure 8. The calculated response/recovery times are as follows: 24 s/42 s for TiO_2_ nanotubes, 23 s/34 s for TiO_2_ NTs/rGO (0.5 wt.%), 18 s/33 s for TiO_2_ NTs/rGO (1 wt.%), and 20 s/35 s for TiO_2_ NTs/rGO (3 wt.%). It is evident that the sensors based on the 1 wt.% rGO doped TiO_2_ NTs/rGO exhibit a significantly faster response time. However, it is worth noting that the response time tends to increase with higher doping amounts of rGO. The relative filling and partial binding of TiO_2_ nanotubes due to rGO doping positively influence gas permeation into the sensing layer as well as electron transfer from TiO_2_ to graphene, which may contribute to a quicker reduction in resistance. Nevertheless, an increase in rGO doping can lead to agglomeration, which reduces the active sites available for gas molecules and consequently diminishes resistance charge. The room temperature NO_2_ gas sensing performances of TiO_2_ NTs/rGO were compared with previous metal-semiconductor oxide/rGO nanocomposites. In Table 1, we can see that in low concentration detection and response/recovery time, TiO_2_ NTs/rGO exhibited better gas sensing performance than most of the previously reported composites.

**Figure 9:**
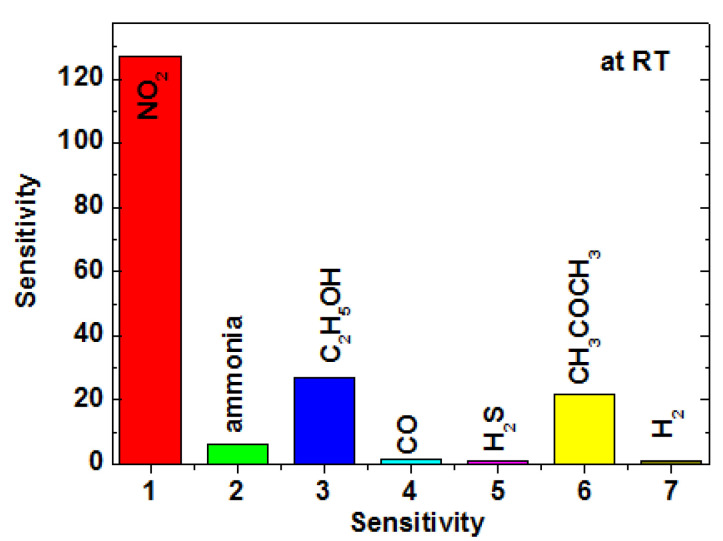
Response of the sensor based on TiO_2_ NTs/rGO nanocomposites to 50 ppm of C_2_H_5_OH, CH_3_OH, H_2_, NH_3_, H_2_S, and NO_2_ at room temperature.

The selectivity of gas sensors is another crucial parameter for real-time applications. As illustrated in Figure 9, the sensor utilizing TiO_2_ NTs/rGO composites (1 wt. %) demonstrated an impressive response to NO_2_, with its value being at least four times greater than that observed for other test gases. This behavior regarding CO, H_2_S, and H_2_ can be attributed to the low operating temperature and relatively low concentration of the detected gases [41]. Based on these findings, it is evident that the sensor employing TiO_2_ NTS/rGO nanocomposites exhibits superior performance in detecting low concentrations of NO_2_ at lower temperatures compared to other detection gases.

**Figure 10:**
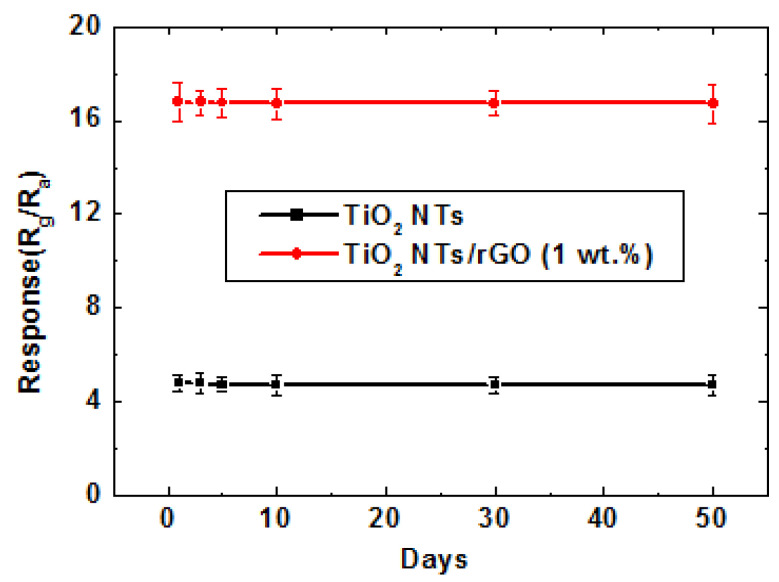
Long-term stability of the sensor based on TiO_2_ NTs and TiO_2_ NTs/rGO nanocomposites to 1 ppm of NO_2_ at room temperature.

The repeatability and long-term stability of the sensor were investigated, providing crucial parameters for practical applications. The long-term stability of the sensors based on TiO_2_ nanotubes (NTs) and TiO_2_ NTs/reduced graphene oxide (rGO) nanocomposites in response to 1 ppm NO_2_ at room temperature is illustrated in Figure 10. The sensor utilizing TiO_2_ NTs/rGO (1 wt.%) exhibited a stable response of 17.2 (17.2 ± 0.3) for 1 ppm NO_2_ at room temperature, demonstrating excellent long-term stability.

### 3.4. Gas Sensing Mechanism of TiO_2_ NTs/rGO Nanocomposites

**Figure 11:**
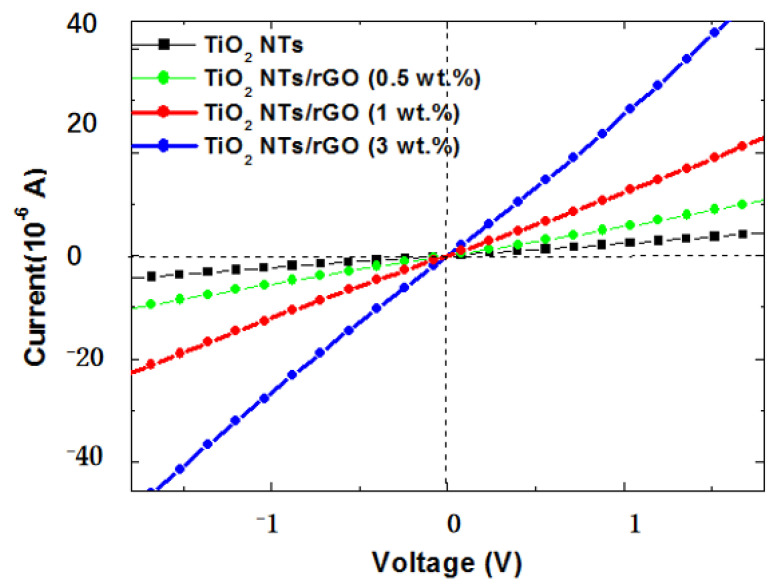
I-V curves of the sensor based on TiO_2_ NTs/rGO nanocomposites with different rGO contents.

The sensing principle of resistance is fundamentally based on the changes in sensor resistance, which occurs due to variations in charge carriers and is closely linked to the amount of chemically adsorbed substances present on the surface of metal oxides. In the case of *n-type* TiO_2_, O_2_ molecules from the atmosphere are adsorbed onto its surface and subsequently transform into O^−^, O_2_^−^, and O^2−^ by capturing electrons from TiO_2_. However, when NO_2_ is introduced, its chemical adsorption further reduces conductivity through an electron capture effect. We have observed that incorporating rGO can significantly enhance the conductivity of these sensors, thereby improving their gas sensing performance at room temperature. As illustrated by the I-V curves shown in Figure 11, it becomes evident that the TiO_2_ NTs/rGO nanocomposite exhibits much lower resistance compared to undoped TiO_2_ nanotubes, which indicates a notable improvement in charge transfer performance. Moreover, with an increase in active sites such as vacancies, defects, and oxygen functional groups, we expect a significant improvement in both the gas adsorption and diffusion rates of NO_2_ molecules on these active surfaces.

**Figure 12:**
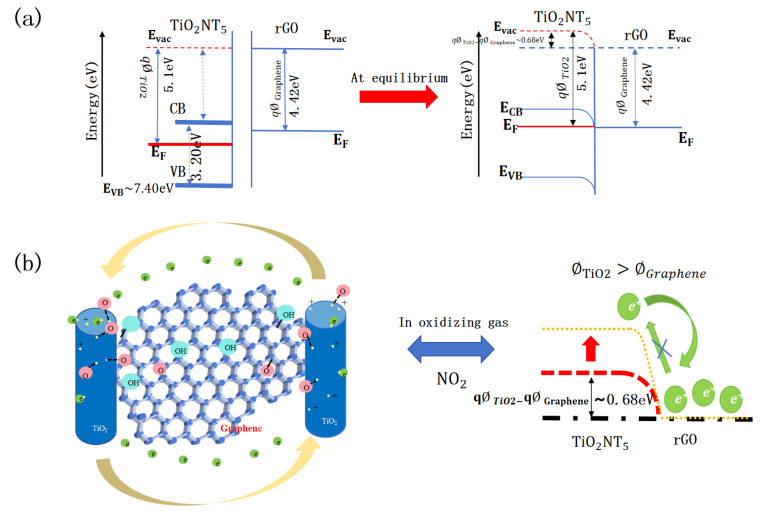
Energy band diagrams for TiO_2_ NTs, rGO and TiO_2_ NTs/rGO heterostructure, where E_VB_, E_F_, E_CB_, E_vac_ represent valence band, Fermi level, conduction band, and vacuum level, respectively. (**a**) The energy band diagrams for TiO_2_ nanotubes, rGO, and their corresponding TiO_2_ NTs/rGO nanocomposites. (**b**) Schematic illustration of electron transfer and sensing mechanism of TiO_2_ NTs/rGO nanocomposites.

Another mechanism contributing to the enhancement of the sensor can be attributed to the formation of a *p-n* heterojunction between *n-type* TiO_2_ and *p-type* rGO. The close contact between these two distinct semiconductor materials allows for the alignment of their Fermi energy levels at the interface, which typically results in charge transfer and the establishment of a charge depletion layer. Figure 12a illustrates the energy band diagrams for TiO_2_ nanotubes, rGO, and their corresponding TiO_2_ NTs/rGO nanocomposites. The work function for TiO_2_ NTs is approximately 5.1 eV, while that of rGO is around 4.42 eV. Upon forming the TiO_2_/rGO heterojunction, an electron accumulation region develops on the surface of TiO_2_ NTs. This charge transfer creates a potential barrier at the heterojunction, leading to bending in both vacuum energy levels and energy bands. The initial electron transfer from TiO_2_ to graphene generates a surface depletion region on TiO_2_, resulting in increased resistance. In ambient air conditions, where no barriers exist between TiO_2_ NTs and graphene, electrons can flow freely from TiO_2_ NTs to graphene. However, upon exposure to NO_2_ gas, there is an increase in potential barrier height at the interfaces between *n-type* TiO_2_ NTs and *p-type* rGO, which makes electron transfer from *p-type* graphene to *n-type* TiO_2_ NTs more challenging and ultimately leads to an increase in sensor resistance (Figure 12b).

## 4. Conclusions

In summary, TiO_2_ nanotube arrays modified by rGO nanosheets were successfully synthesized using a straightforward two-step method. The SEM images reveal that the rGO layer not only partially enveloped the TiO_2_ nanotubes but also established a close connection between adjacent nanotubes, thereby enhancing the efficiency of electron–hole separation. The gas sensing properties of the TiO_2_ NTs/rGO nanocomposites demonstrated high sensitivity to NO_2_ at low operating temperatures, characterized by an enhanced response, relatively short recovery time, and excellent selectivity. The improved gas sensing performance can be attributed to the formation of a *p-n* heterojunction between *n-type* TiO_2_ and *p-type* rGO. Furthermore, due to the presence of rGO sheets, the rate of gas adsorption and diffusion on the active surface of TiO_2_ NTs/rGO nanocomposites was significantly facilitated by an increased number of active sites.

## Figures and Tables

**Table 1:** Comparison of the RT NO_2_ gas sensing performances of our device with those reported in previous literature.

Ref.	Device Structure	Target Gas	Working Temperature (°C)	Response (%)/ppm	Response/Recovery Time (s)
[3]	rGO/CeO_2_	NO_2_	RT	8.2/25	180/260
[4]	In_2_O_3/_rGO	NO_2_	RT	8.25/30	165/235
[5]	Graphene/ZnO	NO_2_	300	9.5/50	145/248
[40]	TiO_2_ NPs/rGO	NO_2_	RT	14.9/100	124/182
This work	TiO_2_ NTs/rGO	NO_2_	RT	19.1/1	18 s/33 s

## Data Availability

Data are contained within the article.
